# Increases in loneliness among Older Americans Act participants during COVID-19

**DOI:** 10.3389/fpubh.2024.1391841

**Published:** 2024-05-01

**Authors:** Heather L. Menne, Jason Osborne, Claire Pendergrast

**Affiliations:** ^1^Department of Sociology and Gerontology, Miami University, Oxford, OH, United States; ^2^Department of Statistics, Miami University, Oxford, OH, United States; ^3^Department of Sociology, Syracuse University, Syracuse, NY, United States

**Keywords:** congregate meals, home-delivered meals, loneliness, Older Americans Act, social ecological model, social engagement

## Abstract

Loneliness is increasingly understood as a public health crisis, and older adults are experiencing particularly severe impacts. Social distancing efforts during the COVID-19 pandemic may have increased loneliness among older adults. Guided by the Social Ecological Model, this study uses two cross-sectional waves of the National Survey of Older Americans Act Participants (NSOAAP) from 2019 and 2021 to expand understanding and identify possible points of intervention to increase social support for vulnerable older adults. Results reveal that while home-delivered meal participants have higher levels of loneliness than congregate meal participants, levels of loneliness did not increase during the COVID-19 pandemic and their loneliness levels did not differ significantly by age, geographic location, or living arrangement. Congregate meal participants’ loneliness increased during the first year of the pandemic, particularly for participants aged 65–74, those living in suburban or rural areas, and those living alone. These findings suggest opportunities for policymakers and aging services providers who seek to increase social engagement among older adults who participate in Older Americans Act (OAA) nutrition programs. The evidence suggests a need for increased social engagement initiatives through OAA programs that prioritize social support for groups who are disproportionately burdened by loneliness.

## Introduction

Loneliness is a growing public health concern, especially for older adults. It is estimated that 43% of adults aged 60 and older are lonely and 24% of adults aged 65 and older are socially isolated (1). In addition, the office of the Surgeon General (2) issued an advisory on loneliness and discussed multiple factors that may increase the risk of loneliness. Empirical literature points to *social isolation* as being an objective measure based on the number of social relationships or social interactions while *loneliness* is understood as a subjective or perceived discrepancy between a person’s desired and actual levels of social engagement and social support (2–4).

Multiple risk factors for increased loneliness in older adults have been identified in previous studies, including age, gender, race, geographic location, and living alone. For example, systematic reviews by Cotterell et al. (5) and Dahlberg et al. (6) identify individual-level factors of age and living alone as associated with social isolation and loneliness. In addition, research by Cudjoe et al. (7) found that having lower levels of income and education were associated with reports of social isolation among older adults in the National Health and Aging Trends Study (NHATS). Their work also identified that older Black and Hispanic adults were less likely to experience social isolation, compared to White older adults (2020). The explanation for these differences may be related to other research using the National Survey of American Life which found that White older adults are more likely to live alone, not have children, and be isolated or have limited contact with members of their religious congregation (8).

A 2023 scoping review by Pickering et al. (4) found conflicting evidence on whether rural geography was associated with more or less social isolation and loneliness among older adults during the COVID-19 pandemic. As way of possible explanation, the authors note inconsistent definitions for *rural* in the studies, with some studies including small towns and other studies limited to remote areas. Separately, a survey of older adults in Canada did not find significant differences in reports of social isolation based on rural vs. urban settings, but income level did have a significant relationship with social isolation (9). In the Canadian study, older adults with low- or middle-income reported ‘often’ feeling isolated more than older adults with high incomes.

Awareness, service delivery, and targeted programs are needed to intervene and address loneliness among older adults (1, 2). Within the United States, Older Americans Act (OAA) services, which are available to any adult aged 60 and over, are a means to increase social connection and reduce loneliness for older adults (10, 11). OAA legislation outlines those services should be prioritized for.

“unserved older individuals with greatest economic need (including low-income minority individuals and older individuals residing in rural areas) and unserved older individuals with greatest social need (including low-income minority individuals and older individuals residing in rural areas)” (12, p. 35).

The services of the OAA, including congregate meals and home-delivered meals, are intended to support the independence of older adults (13, 14). Eligibility for the meal programs is set primarily by states and local service providers, but the OAA does indicate that participants need to be age 60 or older. Typically, home-delivered meal participants are more frail, isolated, and homebound (15).

In response to the COVID-19 pandemic, many steps were taken to adjust how OAA services were provided in light of public health restrictions. OAA service providers acted quickly to expand but also modify their services during the pandemic, and many providers used innovative strategies to support social connection for OAA participants when traditional sources of social support were disrupted (16–18). In addition, in response to the COVID-19 pandemic the American Rescue Plan Act of 2021 allocated $460 million for older adult services and “activities to prevent and mitigate social isolation related to COVID-19” [(19), p. 8]. For example, there was a clear shift in persons served through the home-delivered meal and congregate meal programs. The number of home-delivered meal clients shifted from 883,000 individuals (2019) to 1.4 million (2020) to 1.5 million (2021) (15, 20, 21).

To date no quantitative study has explored changes in social connection around the COVID-19 pandemic for OAA clients. The 2019 and 2021 data collected in the cross-sectional National Survey of Older Americans Act Participants (NSOAAP) provides a unique opportunity to explore how the pandemic impacted the experience of older adults receiving OAA home-delivered and congregate meals. This study expands understanding and points to possible points of intervention to support older adults who often face common risk factors for loneliness (4–6).

### Conceptual model

This study examines the impact of year and characteristics of OAA congregate meal and home-delivered nutrition clients on the subjective outcome of loneliness. This work is grounded in the Social Ecological Model [SEM; (5, 22)] which recognizes nested layers of influence on health outcomes, including loneliness. In line with the report by the office of the Surgeon General (2), the model outlines the importance of individual-, interpersonal- or relationship-, community, and societal- or political- layers on health outcomes.

A systematic review by Dahlberg et al. (6) assessed the literature on older adult risk factors for subjective loneliness over time but did not frame their review with the SEM. An earlier review by Cotterell et al. (5) did use the SEM and focused on the complementary outcome of objective social isolation. In both the reviews, the resulting factors align conceptually with the SEM layers. For example, both reviews identify individual-level factors, and relationship-level factors as associated with social isolation and loneliness (5, 6).

With the SEM as a guide and in light of the current research on loneliness among older adults in general, this initial study focused on individual-level factors addresses the following research questions:

- How lonely were OAA nutrition clients before the COVID-19 pandemic?
- What percentage of OAA nutrition clients were lonely before the COVID-19 pandemic?
- What amount of change in loneliness was experienced by OAA nutrition clients during the COVID-19 pandemic?
- How do individual-level factors of the Social Ecological Model and time explain changes in loneliness for OAA nutrition clients?

## Methods

### Data source

With the exception of 2020, annually the Administration on Aging within the Administration for Community Living conducts the NSOAAP to measure service and program quality and learn more about OAA program participants (14). For this study, we used the 2019 and 2021 NSOAAP- Congregate Meal and Home-delivered Meal modules, which contains responses from 3,592 nutrition services program clients. Respondents answered questions related to demographic and socioeconomic characteristics, well-being, program satisfaction, unmet needs, and service usage.

### Weights

Weights were provided in each data set to reflect the probability sampling methodology used in the surveys, and are used to create a data set that is representative of the population of interest. Applying the weights also inflates the sample size to the population size, which inflates the degrees of freedom and can cause misestimation of standard errors and bias hypothesis tests (23). We scaled each weight to maintain original sample size.

### Combination of data

All four data sets were combined into a single data set containing cases from the 2019 and 2021 surveys of adults receiving home meal delivery and the 2019 and 2021 surveys of adults receiving congregate meals.

### Measures

#### Outcome

Loneliness was measured using the 3-item version of UCLA loneliness scale (24), adapted from the longer 20-item UCLA Loneliness Scale (25, 26). The three-item version was specifically designed for large-scale surveys like the NSOAAP, asking respondents how often they feel that they lack companionship, feel left out, and feel isolation from others (response options 1 = “hardly ever” 2 = “some of the time” and 3 = “often”).

This brief measure strongly correlates with the longer 20-item version (*r* = 0.82) and typically shows reasonable internal consistency [*α* = 0.72; (24)]. In our data, these three items showed strong internal consistency (*α* = 0.82). Responses to these three items were summed to create an index of 3–9 with higher numbers indicating more loneliness. In line with prior literature (27), UCLA loneliness scale scores were also dichotomized to reflect the percentage of “not lonely” (scoring 3–5) and “lonely” (6–9) respondents. The composite score of loneliness and the percentage of people reporting being lonely are outcome metrics used in this analysis because the composite score gives an indication of the amount of loneliness experienced by the respondents and the percentage reflects the prevalence of loneliness in this population. Taken together, these outcomes can inform areas for intervention.

#### Demographics and social factors

Demographic variables are based on self-reported survey responses. Variables included age (60–64 years; 65–74 years; 75–84 years; 85+ years) and gender (1 = male; 0 = female). Because race and ethnicity were asked as unique questions for each category, these were combined to yield a race/ethnicity variable (1 = White, 2 = Black; 3 = Other Race). Social factor variables included geographic location (1 = rural = city, 2 = suburbs; 3 = rural area) and whether respondents live alone (0 = not, 1 = yes).

#### Testing assumptions and data cleaning

In all analyses, assumptions of the analyses were tested, and standardized residuals were evaluated to identify inappropriately influential cases (e.g., outliers). Cases with standardized residuals greater than 2.50 in magnitude were removed from analyses. Degrees of freedom for the analyses vary slightly due to missing data or the removal of outliers.

## Results

### Trends in loneliness from 2019 to 2021

To evaluate whether there were changes in loneliness over time, an ANOVA was computed for the UCLA loneliness scale by year and meal type (Table 1). OAA participants reported significantly higher levels of loneliness in 2021 than 2019 OAA participants, indicating an increase in loneliness during the COVID-19 pandemic [4.79 vs. 4.53; *F*_(1, 3,305)_ = 17.21, *p* < 0.001]. There was also a significant difference based on meal program type. Home-delivered meal participants had significantly higher levels of loneliness than congregate meal participants [5.02 vs. 4.30; *F*_(1, 3,305)_ = 132.7, *p* < 0.001]. There was also a significant interaction of meal and year; home-delivered meal participants’ loneliness remained fairly unchanged from 2019 to 2021 (5.02 vs. 5.03), while congregate meal participants showed marked increases in loneliness during the same time period (4.04 in 2019 vs. 4.56 in 2021).

**Table 1:** UCLA composite loneliness measure and percentage of lonely respondents by year and meal type.

	Home-delivered meals	Congregate meals	Total
2019	5.02 (37.6%)	4.04 (19.2%)	4.53 (28.4%)
2021	5.03 (37.1%)	4.56 (24.8%)	4.79 (31.0%)
Total	5.02 (37.3%)	4.30 (22.0%)	–

UCLA scores range from 3 to 9 with higher numbers indicating higher levels of loneliness. Percent identifying as lonely are in parentheses.

In line with prior literature (27), UCLA loneliness scale scores were also dichotomized to reflect lonely respondents and not lonely respondents. There was no significant main effect of year [*F*_(1, 3,334)_ = 2.60, *p* < 0.11], but there was a significant main effect of meal type. Participants receiving home-delivered meals reported higher rates of loneliness than those receiving meals in congregate settings [*F*_(1, 3,338)_ = 94.17, *p* < 0.001]. There was also a significant interaction of meal and year for the rates of loneliness, showing that the rates for respondents receiving home-delivered meals remained fairly unchanged from 2019 to 2021 while rates of loneliness for those receiving congregate meals increased [*F*_(1, 3,305)_ = 3.85, *p* < 0.05].

### Demographic and social factors impacting loneliness

To explore which factors may impact OAA participant loneliness, a series of interactions were examined within ANOVA, with significant interaction effects indicating that the demographic variable moderated the effect of year and meal type described above. The demographics variables of race and gender were analyzed but did not yield any significant interactions with year and meal (not shown). This means that changes over time did not differ based on gender or race, and that the difference in loneliness between home-delivered meal participants and congregate meal participants was not larger for any gender or racial group.

#### Age

In general, younger respondents, regardless of meal type, reported higher levels of loneliness measured on the UCLA scale [Table 2; *F*_(1, 3,271)_ = 30.58, *p* < 0.001], and there was a significant interaction with year and meal [*F*_(1, 3,271)_ = 3.72, *p* < 0.011]. For example, marked changes in loneliness from 2019 to 2021 for congregate meal participants seems to have been particularly pronounced in the 65–74-year-old group, which saw the largest increase in loneliness amongst those receiving congregate meals (i.e., 3.97 in 2019 to 4.68 in 2021), while those age 65–74 receiving home-delivered meals experienced a reduction in reported loneliness (i.e., 5.40 in 2019 to 5.14 in 2021).

**Table 2:** Loneliness measure by year, meal, and age.

Meal	Year	60–64	65–74	75–84	85+
Home-delivered meals	2019	5.83 (51.5%)	5.40 (45.5%)	4.70 (32.2%)	4.64 (29.4%)
	2021	5.80 (53.7%)	5.14 (37.6%)	4.78 (33.6%)	4.85 (32.2%)
Congregate meals	2019	4.72 (35.1%)	3.97 (13.7%)	3.93 (15.0%)	4.11 (18.2%)
	2021	5.27 (42.3%)	4.68 (27.4%)	4.20 (20.3%)	4.26 (19.8%)
Total		5.40 (45.7%)	4.80 (31.0%)	4.40 (25.3%)	4.46 (24.9%)

UCLA scores range from 3 to 9 with higher numbers indicating higher levels of loneliness. Percent identifying as lonely are in parentheses.

The effects were similar for the rate of loneliness outcome. Younger respondents reported higher levels of loneliness measured on the UCLA scale [45.7, 31.0, 25.3, and 24.9% reporting being lonely for those 60–64, 65–74, 75–84, and over 85, respectively; *F*_(1, 3,271)_ = 19.25, *p* < 0.001], and there was a significant interaction with year and meal [*F*_(1, 3,271)_ = 2.97, *p* < 0.031]. In fact, self-reported loneliness among congregate meal recipients age 65–74 more than doubled from 13.7 to 27.4%, the largest increase among any age group.

#### Geographic location

Whether a respondent lived in an urban, suburban, or rural location was also associated with trends in loneliness, as measured on the UCLA scale [4.53, 4.74, 4.63 for urban, suburban, and rural, respectively; *F*_(1, 3,184)_ = 3.13, *p* < 0.04] and there was a significant interaction with year and meal [*F*_(1, 3,184)_ = 3.60, *p* < 0.028]. As Table 3 shows, suburban respondents tended to report higher levels of loneliness, and home-delivered meal recipients similarly reported higher levels of loneliness. Looking at changes over time, levels of loneliness among urban OAA participants remained fairly consistent from 2019 to 2021. Urban home-delivered meal recipients tended to retain higher levels of loneliness, and urban respondents receiving congregate meals tended to remain fairly constant at lower levels of loneliness. Suburban respondents tended to show the strongest changes from 2019 to 2021. Suburban home-delivered meal recipients showed a marked increase in loneliness, but those receiving meals in a congregate setting had nearly a full 1-point average increase in levels of loneliness over time (i.e., 4.03 in 2019 to 4.99 in 221). Rural respondents showed mixed patterns, with home-delivered meal recipients showing decreases in loneliness, while rural respondents receiving congregate meals showed marked increases in loneliness.

**Table 3:** Loneliness measure by year, meal, and location of home.

Meal	Year	Urban	Suburb	Rural
Home-delivered meals	2019	5.07(37.0%)	4.76(33.8%)	5.12(40.2%)
	2021	4.99 (36.6%)	5.17 (41.4%)	4.87 (30.7%)
Congregate meals	2019	4.05 (16.0%)	4.03 (15.1%)	3.98 (17.2%)
	2021	4.02 (13.4%)	4.99 (32.8%)	4.53 (26.9%)
Total		4.53 (25.8%)	4.74 (30.8%)	4.63 (28.8%)

UCLA scores range from 3 to 9 with higher numbers indicating higher levels of loneliness. Percent identifying as lonely are in parentheses.

The effects were similar for the dichotomous loneliness variable. Younger respondents reported higher levels of loneliness measured on the UCLA scale [25.8, 30.8, and 28.8% for urban, suburban, and rural, respectively; *F*_(1, 3,184)_ = 3.32, *p* < 0.036], and there was a significant interaction with year and meal type [*F*_(1, 3,184)_ = 4.51, *p* < 0.011]. There was a clear increase in the percentage of suburban home-delivered meal recipients reporting being lonely, but those receiving meals in a congregate setting more than doubled the percent reporting loneliness.

#### Living alone

In general, living with another person was associated with less loneliness than not living with someone [*F*_(1, 3,281)_ = 64.16, *p* < 0.001]. There was a significant three-way interaction between this variable and meal and year [*F*_(1, 3,281)_ = 12.79, *p* < 0.001]. Those who received their meals at home and lived alone had the highest levels of loneliness, with little change in their high levels of loneliness over time. Those who received congregate meals and lived alone showed much lower loneliness in 2019, but in 2021, they had experienced a substantial increase in loneliness (Table 4).

**Table 4:** Loneliness measure by year, meal, and living alone.

Meal	Year	Live alone	Live with another
Home-delivered meals	2019	5.26 (41.9%)	4.69 (31.9%)
	2021	5.15 (41.8%)	4.90 (31.9%)
Congregate meals	2019	4.22 (20.3%)	3.90 (13.8%)
	2021	4.96 (35.3%)	4.09 (13.1%)
Total		4.90 (35.6%)	4.40 (23.4%)

UCLA scores range from 3 to 9 with higher numbers indicating higher levels of loneliness. Percent identifying as lonely are in parentheses.

The effects were similar for the rate of loneliness outcome. An interesting pattern is observed where the rates of loneliness remain consistent over time with the meals type and whether people live alone, with one exception. While 20% of congregate meal participants who live alone reporting being lonely in 2019, this percentage increased to 35% for 2021. In contrast, a consistent 42% of home-delivered meal participants who lived alone reporting being lonely, and this was the highest percentage among the meal types when considering whether people lived alone.

## Discussion

With the decreases in social interaction that occurred with the COVID-19 pandemic, there was concern about whether older adults’ loneliness or lack of social engagement may present serious and long-term health risks (2). Among the entire sample of older adults in this study, there was not a significant change in the percentage of older adults reporting loneliness between 2019 (28.4%) and 2021 (31.0%); however, there were differences over time based on the meal program type. For home-delivered meal participants, the percentage reporting loneliness was consistently at 37%, but for congregate meal participants there was an increase from 19.2% in 2019 to 24.8% in 2021 of reporting loneliness. This difference in the experience of meal program participants may be attributed to the primary reasons people use the different programs and whether those motivations were still met being met in the post-pandemic experience. For example, home-delivered meals are typically provided to older adults who are more frail and more likely to be homebound than congregate meal participants (15). Relatedly, research has documented that the primary reason one attends the OAA congregate meal program is socialization (28). The pandemic brought about changes to the congregate meal program such that the typical in-person programming of meals and education was paused and more home-delivered or grab-and-go meals were provided, which in turn limited or changed the socialization opportunity for participants (19).

To understand points of intervention for social engagement in these meal programs, this initial analysis focused on demographic and social factors of the individual program participants. While race and gender variables did not show significant interactions with year and meal type, analyses by age category revealed important considerations for increasing engagement and awareness. Younger OAA nutrition clients experienced more loneliness than older clients, and this difference was evident in 2019 and 202. This may indicate that younger older adults, those age 60–64, are using services when they were expecting to still be in the work force. They may be facing more health conditions or disabilities compared to others in their age category, or they may be attending a congregate setting that does not hold social activities they enjoy. Local service providers might explore how younger older adults can be more engaged through home-delivered or congregate meals. For example, the work by Thomas et al. (11) documents how home-delivered meal participants have lower levels of loneliness compared to similar older adults not receiving home-delivered meals, and one explanation is the benefits of the (albeit oftentimes limited) interaction between the meal recipient and the meal delivery driver (11). Some participants may develop friendships with their delivery driver, whereas other participants may only see their delivery driver for the few moments it takes to hand over the meal.

Results related to social factors of geographic location and whether a person lives alone also point to areas for possible intervention to support older adults using OAA nutrition programs. For suburban and rural congregate meal clients, there was a distinct increase in loneliness scores and the percentage reporting being lonely between 2019 and 2021. These results may be related to the distance a participant needs to travel to participate in a congregate meal, which may have increased if settings closed or reduced hours. In addition, friendships formed pre-pandemic in congregate settings may have shifted if participants are not attending in the same location or on the same day in 2021. Conversely, there were 10% fewer rural-living home-delivered meal clients reporting being lonely in 2021 compared to 2019. Due to the cross-sectional nature of the NSOAAP data, we cannot make causal claims; however, with the pandemic there was an increased emphasis on delivering meals to home-bound older adults, and this may have impacted how participants experienced those meal deliveries and thus they reported less loneliness in 2021.

People living alone, regardless of year or meal program, had higher loneliness scores and more of them reported being lonely. For the most part, living alone or with another person had little association with loneliness among OAA nutrition program participants. The levels of loneliness and percent of people reporting being lonely were higher for home-delivered meal participants who lived alone (compared to home-delivered meal clients who did not live alone), but the levels were consistent among the two groups of home-delivered meal clients over time. This may suggest that the pandemic did not change the experience for home-delivered meal clients, and further investigation might explore whether living arrangement has a buffering effect on loneliness for these clients.

The results of loneliness over time for congregate meal participants by living arrangement tell a different story from the experience of home-delivered meal participants. Minimal changes in loneliness were seen for congregate meal participants who do not live alone, but there was a clear increase from 2019 to 2021 in levels of loneliness and the percentage reporting being lonely for congregate meal participants. This result is similar to what was observed in relation to geographic location with an increase in loneliness for suburban and rural congregate meal participants over time. The social engagements and connections fostered pre-pandemic through congregate meal participation may have been stymied during the 2021 data collection. With additional waves of data, it will be important to observe whether levels of loneliness among congregate meal participants return to pre-pandemic levels.

Secondary analyses of cross-sectional survey data do possess some limitations that require acknowledgment. First, all variables used in the analyses are based on self-report by program participants. Other self-reported measures for loneliness could have been used in the survey, but it understandable that ACL chose to collect the UCLA Loneliness Scale because it is commonly used with older adults, is short and limits burden on respondents, and is associated with objective measures of social isolation in older adult (11). Second, when there are complex samples being analyzed, appropriate application of weights typically leads to more accurate estimation of population parameters and more defensible inferences where simple random sampling is not desirable or feasible. This data set is the result of probabilistic sampling, and as such, failure to appropriately weight the data prior to analysis could yield biases [see, e.g., (29–31)]. One potential undesirable effect of applying weights is that the sample size typically inflates to the population, which inflates degrees of freedom used for inferential statistics inappropriately. Thus, we scaled weights to produce representative estimates while preserving sample size [e.g., normalized or relative weights, (32, 33)]. The other potential for issues is to use the wrong weighting scheme. Complex (especially longitudinal) surveys can have many different weights that are used for different reasons. In this case, the agency that provided the data file also provided the appropriate weights, which we utilized as noted above.

Taken together, these results yield evidence which can be used by policymakers and providers who seek to increase social engagement among OAA clients. The evidence points to the need for increases in social engagement initiatives for OAA programs and it highlights the need for prioritizing social engagement initiatives with groups who are disproportionately burdened by social isolation and loneliness (2). While this is a preliminary study, the results highlight key individual-level factors such as age, geographic location, and living arrangement which are of paramount importance to nutrition program providers. The use of the Social Ecological Model, and specifically variables measuring interpersonal, community, and societal factors may uncover associations between loneliness, time, and health conditions, functional abilities, other family or formal supports, or accessibility of needed services.

## Data availability statement

Publicly available datasets were analyzed in this study. This data can be found at: https://agid.acl.gov/.

## Ethics statement

The studies involving humans were approved by Miami University Institutional Review Board. The studies were conducted in accordance with the local legislation and institutional requirements. Written informed consent for participation was not required from the participants or the participants’ legal guardians/next of kin in accordance with the national legislation and institutional requirements.

## Author contributions

HM: Conceptualization, Writing – original draft, Writing – review & editing. JO: Conceptualization, Data curation, Formal analysis, Writing – original draft, Writing – review & editing. CP: Conceptualization, Writing – review & editing.
